# Case Report: Patient with deficiency of ADA2 presenting leukocytoclastic vasculitis and pericarditis during infliximab treatment

**DOI:** 10.3389/fped.2023.1200401

**Published:** 2023-06-14

**Authors:** Diana Simão Raimundo, Ana Isabel Cordeiro, João Parente Freixo, Marta Valente Pinto, Conceição Neves, João Farela Neves

**Affiliations:** ^1^ Pediatrics Department, Hospital do Divino Espírito Santo, Ponta Delgada, Portugal; ^2^ Primary Immunodeficiencies Unit, Hospital Dona Estefânia, Centro Hospitalar Universitário de Lisboa Central, Lisboa, Portugal; ^3^ Centro de Genética Preditiva e Preventiva, Instituto de Biologia Molecular e Celular, Instituto de Investigação e Inovação em Saúde, Porto, Portugal; ^4^ Egas Moniz Center for Interdisciplinary Research (CiiEM); Egas Moniz School of Health & Science, Caparica, Almada, Portugal; ^5^ NOVA Medical School, Faculdade de Ciências Médicas, NMS, FCM, Universidade NOVA de Lisboa, Lisboa, Portugal; ^6^ CHRC, NOVA Medical School, Faculdade de Ciências Médicas, NMS, FCM, Universidade NOVA de Lisboa, Lisboa, Portugal

**Keywords:** DADA2, pericarditis, leukocytoclastic vasculitis, infliximab, anti-TNF-α, adenosine deaminase 2 (ADA2)

## Abstract

Deficiency of adenosine deaminase 2 (DADA2), first reported in 2014, is a disease with great phenotypic variability, which has been increasingly reported. Therapeutic response depends on the phenotype. We present a case of an adolescent with recurrent fever, oral aphthous ulcers, and lymphadenopathy from 8 to 12 years of age and subsequently presented with symptomatic neutropenia. After the diagnosis of DADA2, therapy with infliximab was started, but after the second dose, she developed leukocytoclastic vasculitis and showed symptoms of myopericarditis. Infliximab was switched to etanercept, with no relapses. Despite the safety of tumor necrosis factor alpha inhibitors (TNFi), paradoxical adverse effects have been increasingly reported. The differential diagnosis between disease new-onset manifestations of DADA2 and side effects of TNFi can be challenging and warrants further clarification.

## Introduction

Deficiency of adenosine deaminase 2 (DADA2) is an autosomal recessive disease first described in 2014 by two independent groups as a monogenic vasculitis syndrome resembling polyarteritis nodosa (PAN) (1, 2). It is caused by mutations in the adenosine deaminase 2 (ADA2) gene, formerly CECR1, on chromosome 22q11.1, encoding the enzyme ADA2 (1, 2). With now more than 300 DADA2 patients described, it has been understood that they group in three major phenotypes: vasculopathy (the predominant phenotype), hematologic disorders, and immunodeficiency. Overlapping manifestations occur frequently (3–5).

The function of ADA2, and therefore the pathophysiology of DADA2, has still not been completely elucidated (5). ADA2 regulates the extracellular concentration of adenosine and exerts an anti-inflammatory response during acute conditions (5). Conversely, chronically increased extracellular adenosine levels, as seen in DADA2, promotes a proinflammatory environment and thus endorses chronic inflammation with tissue injury (5, 6). Proposed pathophysiology of DADA2, mainly respecting vasculopathy, includes polarization from the M2 macrophage subtype to the proinflammatory M1 subtype, chronic neutrophil activation and dysregulation of NETosis (formation of neutrophil extracellular traps), leading to endothelial damage and inflammation (3, 5–7). Vasculopathy phenotype responds effectively to anti-inflammatory therapy (3, 5), and tumor necrosis factor alpha (TNF-α) blockade is usually the therapy of choice (5, 6, 8). Hematological manifestations of DADA2 are mostly due to bone marrow hypofunction resulting in erythroblastopenia, leukopenia, neutropenia, and thrombocytopenia and to autoimmune cytopenias (9). In hematologic phenotype, hematopoietic stem cell transplantation (HSCT) is the ultimate treatment (6). Nevertheless, TNF-α inhibition is also usually offered to patients with the latter phenotype, to eventually prevent the potential risk of involvement of the central nervous system (5, 6).

Leukocytoclastic vasculitis, pericarditis, and myopericarditis are included in the vastness of clinical manifestations of DADA2 (3). However, these entities have also been reported as side effects of TNF-α inhibitors (TNFi) (10, 11).

## Case description

**Figure 1:**
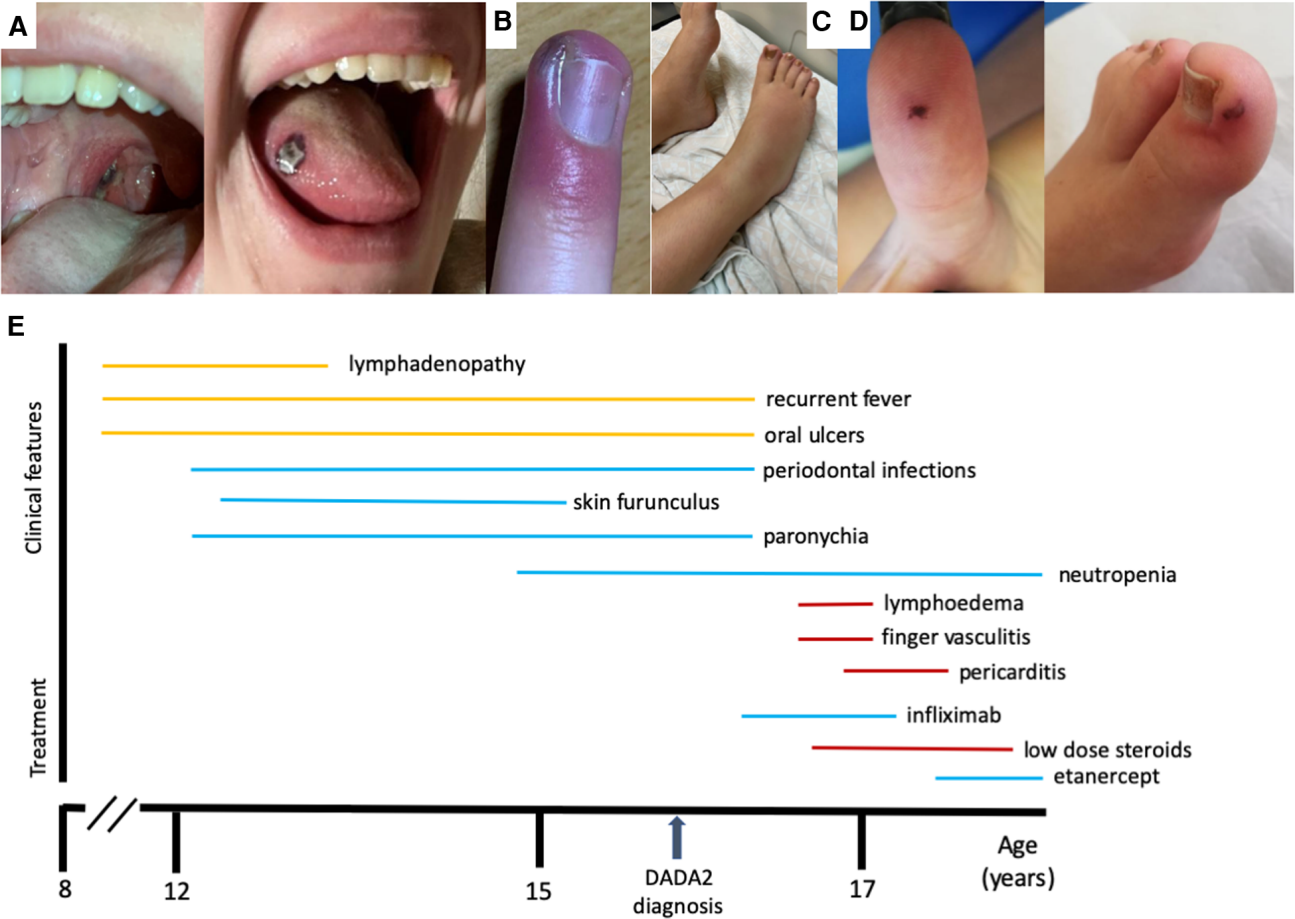
(**A**) Oral ulcer. (**B**) Paronychia. (**C**) Leg lymphedema. (**D**) Vasculitic lesions. (**E**) Timeline for the clinical manifestations and treatments of the patient.

We describe the case of a 16-year-old Caucasian female born to non-consanguineous parents, from an uneventful pregnancy and delivery, with unremarkable family history. Since the age of 8 years old, she experienced monthly episodes of self-limited recurrent fever, oral aphthous ulcers, and lymphadenopathy, lasting for 1 week. There were no other symptoms or signs, namely, abdominal pain, rash, or arthralgia. Her physical examination was normal, with no dysmorphisms or growth failure. There were also no records of severe infections or chronic diarrhea. Since the age of 12, the patient also presented recurrent periodontal infections, as well as frequent episodes of skin furuncles and paronychia, frequently needing antibiotics (Figures 1A,B).

**Figure 2:**
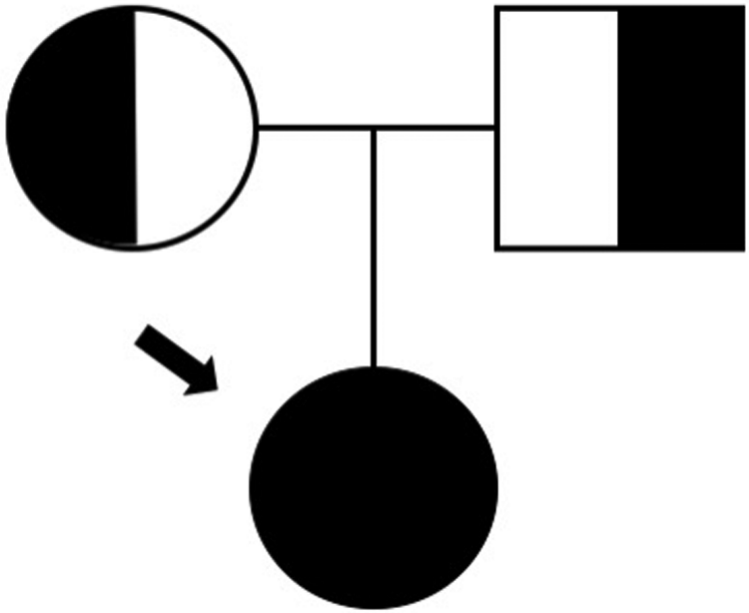
Family tree.

By the age of 15, severe neutropenia (40 cells/μl) was found in blood analysis and confirmed in repeated blood counts. Hemoglobin, platelets, and other leukocyte series were consistently within the normal range. The patient was referred to the primary immunodeficiency clinic at the pediatric hospital. Autoimmune neutropenia was excluded by negative IgG and IgM anti-neutrophil cytoplasmic antibodies. Immunoglobulin levels and vaccine responses were within the normal range. Immunological phenotyping revealed a CD3+ CD8+ lymphocytosis caused by an expansion of terminally differentiated CD8+ T cells, as well as a T + TCRgd+ lymphocytosis and mild NK cell lymphopenia. There were no auto-antibodies nor chronic viral infections (Table 1). A whole exome sequencing (WES)-based customized 115 gene virtual panel for neutropenia and autoinflammatory diseases identified two previously reported in DADA2 patients’ pathogenic variants in the ADA2 gene: c.1358A > G and c.506G > A. We then performed measurement of plasma ADA2 enzymatic activity, which was found to be nearly absent (Table 1); the patient's parents were also studied and were found to be healthy carriers (Figure 2), leading to the diagnosis of DADA2 caused by compound heterozygosity in the ADA2 gene.

**Table 1:** Results of immunologic investigation.

Immunologic investigation	Results	Reference
Immunoglobulin G (mg/dl)	1,700	650–1,850
Immunoglobulin A (mg/dl)	455	70–400
Immunoglobulin M (mg/dl)	165	40–230
ANA	Positive	—
Anti-dsDNA antibody	Negative	—
ENA	Negative	—
Anti-neutrophil antibody IgM, IgG	Negatives	—
Vaccine responses		
Diphtheria (UI/ml)	0.22	↓
Tetanus (UI/ml)	0.88	
Lymphocytes	**4,399**	**1,111–4,202**
B cells [CD19+ (cells/μl)]	**140**	**75–627**
Pre-germinal	92.3	38.7–491
Post-germinal	48.2	26.8–232
Unswitched	22	1 1– 113
Switched	25.9	14.3–130
T cells [CD3+ (cells/μL)]	**4,209**	**898–2,910**
CD4+/CD8− (cells/μl)	**534**	**525–1,782**
Naïve	228	170–1,192
Central memory	291	207–649
Effector memory	14.8	13.4–132
Effector TD	0.33	0–53.2
CD8+/CD4− (cells/μl)	**2,362**	**236–1,143**
Naïve	165	61.7–727
Central memory	588	69.7–422
Effector memory	53.8	2.9–53
Effector TD CD27+ d	493	1.8–91.7
Effector TD CD27−	1,063	0.8–242
T TCRgd+ (cells/μl)	**1,271**	**36.1–391**
T TCRgd− (cells/μl)	**0.84**	**6.5–53.8**
NK CD3−/CD16 56+ (cells/μl)	**45.5**	**111–962**
T cells TCRαβ CD4−/CD8− (%)	1	>6%
IL-10 (pg/ml)	30.7	>20
sFasL	421	>200
ADA2 activity (U/L)	**1.3**	**>5.15**

ANA, anti-nuclear antibody; ENA, extractable nuclear antigen antibodies.

The values in bold are outside the range of normality values, marked in front of each one as reference values.

After DADA2 diagnosis, HSCT was proposed but declined by the family. Intravenous TNFi infliximab was started (5 mg/kg, 6/6 weeks). Interestingly, no episodes of skin furuncles, paronychia, or periodontal infections have occurred since, despite persistent neutropenia.

Four weeks after the second infliximab infusion, the patient developed bilateral tibiotarsal inflammation, which temporarily improved with ibuprofen, but progressed to bilateral lymphedema of leg and foot (Figure 1C). Prednisolone 0.75 mg/kg/day was administered for 3 days that led to complete resolution. One week later, she developed retrosternal pain, as well as severe acute pain in the hands and feet preceding the appearance of lesions of vasculitis in both thumbs and toes (Figure 1D). Skin biopsy of the finger lesions showed vasculitis of the vessels of the superficial dermis, with neutrophil and erythrocyte extravasation, as well as leukocytoclasia and epidermal necrosis. These histopathological findings were compatible with leukocytoclastic vasculitis. Cardiac enzymes, d-dimers, and electrocardiogram (ECG) were unremarkable. Thorax CT angiography revealed no signs of pulmonary thromboembolism, aortitis, or vasculitis of other vessels. Prednisolone was restarted in dose of 1 mg/kg/day for 1 week with clinical improvement of the hand and feet complaints. However, the retrosternal pain persisted, mostly in deep breathing. Inflammatory markers were mildly elevated (C-reactive protein 24 mg/L, sedimentation rate 30 mm). Toponin levels and Creatine Kinase-Myocardial Band (CK-MB) were persistently normal. Echocardiogram showed no major alterations, but ECG revealed *de novo* changes in repolarization, suggestive of acute pericarditis. The cardiac magnetic resonance corroborated pericardial inflammation and also revealed myocardial fibrosis in septum and lateral wall, suggestive of chronic myopathy. No infectious cause of pericarditis was identified. She had received the COVID-19 vaccine 1 year before, and SARS-CoV2 PCR was negative. Antibodies-to-infliximab were absent, and the test for TNF-α levels were not performed. Extensive autoimmune profiling was also negative. The patient was treated with ibuprofen 600 mg every 8 h and colchicine 0.5 mg twice a day and tapered prednisolone, with complete clinical resolution over the following 2 months.

As a rare secondary effect of infliximab could not be ruled out, infliximab was stopped after these episodes of limb ischemia and cardiac lesion; subcutaneous etanercept was started 50 mg once a week, and the patient has been stable since then (Figure 1E). Interestingly, after starting TNF inhibition, she has not had recurrent fever, mouth ulcers, or skin infections, despite persistent neutropenia.

## Discussion

Although its monogenic etiology, DADA2 manifests with high variability, from mild symptoms to fatal vasculitis (3, 8). Our patient initially presented with recurrent fever and lymphadenopathy, and since the age of 12, she also presents a hematologic phenotype, with neutropenia causing the clinical manifestations of frequent cutaneous and perioral infections. Recurrent non-infectious fever, as presented by our patient, emerges as the most frequent autoinflammatory manifestation (51.8% of DADA2 patients) (5). Neutropenia have been mostly reported in frequencies around 15%, and even within hematologic phenotype, predominant neutropenia is very uncommon (12, 13). A recent Saudi cohort of 21 patients from 14 families, however, encountered neutropenia in 76% of patients and described hematological phenotype as predominant; nevertheless, it is noteworthy that mild neutropenia is a common clinical problem in that region (14). Oral aphthous lesions are an uncommon finding in overall DADA2 patients (12, 13, 15).

It has become evident that therapeutic response depends on the phenotype (3, 5). ADA2 is involved in both innate and adaptive immunity cell lineages (1). One of the physiologic roles of ADA2 is to maintain the balance of myeloid lineage cells and their differentiation (2). Insufficient or absent enzymatic activity of ADA2 leads to reduced conversion of adenosine to inosine and consequent accumulation of adenosine in the extracellular space (1, 2). This change in adenosine pathways that occur in DADA2 leads to chronic neutrophil activation and NETosis dysregulation. A pathological increased polarization from the M2 macrophage subtype to the proinflammatory M1 subtype occurs in DADA2, with consequent hypersecretion of inflammatory cytokines, especially TNF-α (1, 2). TNF-α acts on neutrophils contributing to maintain their chronic activation state. NETosis dysregulation also contributes to the M1 polarization, and consequently increases macrophage release of TNF-α (1, 2). TNF-α is one of the main mediators of vascular inflammation. Increased TNF-α has been found both in peripheral blood and in affected tissues from DADA2 patients during active disease (1, 2). This explains the efficacy of the TNF-α blockade in DADA2 patients, especially those presenting vasculitic phenotypes (2). TNF-α inhibition has shown remarkable efficacy in improving features of systemic inflammation and in preventing stroke (5, 6, 8). There is insufficient data to support one TNFi over another, with etanercept, adalimumab, and infliximab being the most commonly used (3). Anti-drug antibodies appear commonly during therapy with anti-TNF inhibitors and may cause allergic reactions or loss of drug efficacy. This risk is least common with etanercept (receptor fusion protein) and most common with infliximab (chimeric antibody) and adalimumab (humanized antibody) (16). The use of other cytokine inhibitors, such as IL-1 blockers and IL-6 blockers, has been anecdotally used with heterogeneous results (5). Corticosteroids can be useful in the acute phase of the disease and flares (9).

In contrast, patients with predominant hematologic phenotypes do not seem to respond satisfactorily to TNFi or corticosteroids, and variable responses have been documented when using other immunosuppressive agents (azathioprine, mycophenolate mofetil, cyclosporine, and anti-thymocyte globulin) (6, 9). Ultimately, the definitive treatment for hematological and immunological manifestations is HSCT, which also has been reported to restore ADA2 activity (6, 9). An international survey on the outcome 18 months after HSCT in DADA2 patients reported effective treatment also in inflammatory manifestations (such as vasculitis, arthritis, and PAN) (17).

Until recently, only two DADA2 patients have been reported to present overlapping inflammatory and hematologic phenotypes: Al-shaikh et al. reported the case of an 18-month-old DADA2 patient presenting recurrent fever, persistent neutropenia, oral aphthous ulcers, and skin abscess, which did not respond to IV methylprednisolone, Intravenous immune globulin (IVIG), Granulocyte colony-stimulating factor (G-CSF), or infliximab. The febrile episodes responded to therapy with IL-1 inhibitor, anakinra, but the patient eventually perished due to persistent perirectal mass bleeding and multiple organ failure (18). A patient reported by Göschl et al. presented neutropenia, recurrent fever, and oral ulcers and later hypogammaglobulinemia, from 8 to 24 years of age. After DADA2 diagnosis, TNFi infliximab in combination with IVIG and prednisolone rapidly led to complete resolution of the symptoms, including the neutropenia (15). Similarly, after starting TNFi, our patient had complete resolution of the recurrent fever, as well as all the symptoms related to neutropenia (recurrent mouth ulcers, periodontitis, and skin infections) despite persistent neutropenia. This is probably related to the different effects that the TNF-α blockade has on DADA2, but the relationship between neutropenia, inflammation, and anti-TNF drugs deserves further clarification. Recently, a cohort of 60 patients described another 6 patients presenting an inflammatory/hematologic phenotype and 21 presenting inflammatory/hematologic and immune phenotype (19).

Unexpectedly, 10 weeks after starting TNFi, our patient developed leukocytoclastic vasculitis with ischemic lesions of the toes that responded very well to steroid treatment. This was followed by myopericarditis, for which no infectious cause was identified. She responded very well to colchicine and ibuprofen. Due to some rare cases of infliximab-related pericarditis, a switch to etanercept was performed, as there is evidence of favorable results with this strategy (10, 20–22).

TNFi are increasingly used for the treatment of inflammatory and rheumatoid diseases (23). Common adverse effects of infliximab include severe infections, hepatotoxicity, infusion reactions, serum sickness-like disease, and lymphoma (24). Paradoxical inflammation and autoimmune reactions have been increasingly identified during treatment with TNFi (23, 25). Vasculitis accounts for 31% of autoimmune diseases secondary to TNFi therapies, mostly being leukocytoclastic vasculitis (25), similar to what was found in our patient’s skin biopsy. We found no reports of direct autoimmune side effects of TNFi in patients with DADA2. Most reported cases occur in the treatment of Crohn's disease, rheumatoid arthritis, and spondyloarthritis (25, 26).

Pericarditis is a very rare adverse reaction when using infliximab therapy, with less than a dozen cases reported (20). In those, pericarditis occurred from 12 days to years after starting infliximab, and most reported cases come from adults treated for Crohn's disease (10, 20–22). Infliximab was commonly replaced by other TNFi, with no recurrence of pericarditis (10, 20–22). In one case, continuing infliximab led to drug-induced systemic lupus erythematosus (SLE), which resolved after stopping this therapy (21). We found no cases of pericarditis secondary to TNFi in DADA2 patients.

The exact mechanism by which infliximab causes pericarditis is not clearly understood (10). Pericarditis may be a manifestation of serum sickness-like reaction (type III hypersensitivity), a known side effect of infliximab (10). On the other hand, TNFi can induce inflammation and autoimmunity induced both directly by inducing inflammatory gene expression and indirectly by inducing cell death (11).

Pericarditis, myocarditis, and myopericarditis have been described as part of the DADA2 spectrum (3), as has the leukocytoclastic vasculitis. Although very unexpected, it cannot be excluded that myopericarditis and ischemia of the toes could also be explained as manifestations of DADA2, even presenting 10 weeks after the initiation of infliximab. However, the development of the first episode of vasculitis after starting therapy with infliximab would seem to be paradoxical, since treatment with TNFi reduces in inflammation and reestablishes integrity of endothelium (27).

In summary, we present the case of a DADA2 patient with overlapping autoinflammatory and hematologic phenotypes that were controlled with TNF-α inhibition, which developed pericarditis and leukocytoclastic vasculitis 10 weeks after the start of TNFi. An adverse reaction to infliximab was suggested by the temporal association between starting this therapy and the onset of symptoms, as well as between stopping the medication and complete clinical resolution without recurrence. DADA2 remains an example of the beautiful complexity of the immune system, as a single change leads to a wide range of consequences. The treatment still remains somewhat empirical. As long as the mechanisms of this disease are not known in detail, we will continue to be liable to unpredictable consequences of our clinical action.

## Data Availability

The original contributions presented in the study are included in the article, further inquiries can be directed to the corresponding author.
